# Mosaic variants detectable in blood extend the clinicogenetic spectrum of *GLI3*-related hypothalamic hamartoma

**DOI:** 10.1016/j.gimo.2023.100810

**Published:** 2023-04-20

**Authors:** Timothy E. Green, Mark F. Bennett, Ilka Immisch, Jeremy L. Freeman, Karl Martin Klein, John F. Kerrigan, Lata Vadlamudi, Erin L. Heinzen, Ingrid E. Scheffer, A. Simon Harvey, Felix Rosenow, Michael S. Hildebrand, Samuel F. Berkovic

**Affiliations:** 1 Epilepsy Research Centre, Department of Medicine, The University of Melbourne, Austin Health, Heidelberg, VIC, Australia; 2 Population Health and Immunity Division, The Walter and Eliza Hall Institute of Medical Research, Parkville, VIC, Australia; 3 Department of Medical Biology, University of Melbourne, Parkville, VIC, Australia; 4 Epilepsy Center Hessen and Department of Neurology, Philipps University Marburg, Marburg (Lahn), Germany; 5 Department of Neurology, The Royal Children’s Hospital, Parkville, VIC, Australia; 6 Murdoch Children’s Research Institute, The Royal Children’s Hospital, Parkville, VIC, Australia; 7 Departments of Clinical Neurosciences, Medical Genetics and Community Health Sciences, Hotchkiss Brain Institute & Alberta Children’s Hospital Research Institute, Cumming School of Medicine, University of Calgary, AB, Canada; 8 Epilepsy Center Frankfurt Rhine-Main and Department of Neurology, Goethe University and University Hospital Frankfurt, Frankfurt am Main, Germany; 9 LOEWE Center for Personalized Translational Epilepsy Research (CePTER), Goethe University Frankfurt, Frankfurt am Main, Germany; 10 Division of Pediatric Neurology, Barrow Neurological Institute, Phoenix Children’s Hospital, Phoenix, AZ; 11 University of Queensland Centre for Clinical Research, University of Queensland, Brisbane, QLD, Australia; 12 Department of Neurology, Royal Brisbane and Women’s Hospital, Brisbane, QLD, Australia; 13 Eshelman School of Pharmacy, Division of Pharmacotherapy and Experimental Therapeutics, Department of Genetics, University of North Carolina, Chapel Hill, NC; 14 Department of Paediatrics, University of Melbourne, Royal Children’s Hospital, Parkville, VIC, Australia; 15 The Florey Institute of Neuroscience and Mental Health, Parkville, VIC, Australia

**Keywords:** *GLI3* gene, Hypothalamic hamartoma, Mosaicism, Pallister-Hall syndrome, Polydactyly

## Abstract

**Purpose:**

Hypothalamic hamartoma (HH) can be syndromic (eg, Pallister-Hall syndrome [PHS], HH, and mesoaxial polydactyly) or nonsyndromic. Most PHS cases have germline variants in *GLI3*, but a minority remain unresolved. Some nonsyndromic HH cases have *GLI3* mosaic variants in the brain. PHS and nonsyndromic HH are regarded as 2 separate *GLI3*-related disorders, clinically and genetically. Here, we searched for mosaic variants in unsolved cases.

**Methods:**

High-depth exome sequencing was performed on leukocyte-derived DNA in 1 unsolved PHS and 25 nonsyndromic HH cases. We searched for mosaic variants in *GLI3* and other HH-associated genes. Mosaic variants were confirmed by droplet-digital polymerase chain reaction.

**Results:**

The PHS case had a *GLI3* stop-gain variant c.2845G>T; p.(Glu949Ter) at 6.9% variant allele fraction (VAF). Two nonsyndromic cases had *GLI3* variants—a stop-gain (c.2639C>A; p.(Ser880Ter), VAF 3.7%) and a frameshift (c.3326_3330del; p.(Glu1109AlafsTer18), VAF 7.8%). The nonsyndromic patient with 3.7% VAF in blood had 35.8% VAF in HH tissue. He had a vestigial extra digit removed adjacent to his left fifth finger.

**Conclusion:**

*GLI3* mosaicism is associated with a phenotypic spectrum from PHS to HH with subtle extra PHS features, to isolated nonsyndromic HH. High-depth sequencing permits detection of low-level mosaicism, which is an important cause of both syndromic and nonsyndromic HH.

## Introduction

Hypothalamic hamartoma (HH) is a benign congenital lesion of the hypothalamus. The most important gene associated with HH is the sonic hedgehog (SHH) pathway master regulator and transcription factor, *GLI3.* It is the common cause of Pallister-Hall syndrome (PHS; OMIM 146510) with autosomal dominant inherited or *de novo* pathogenic variants.^1^^,^^2^ The key features of PHS are HH and mesoaxial polydactyly, with variable features including limb malformation, bifid epiglottis, and imperforate anus.^3^

More commonly, HH is nonsyndromic, and it is usually recognized in epilepsy cohorts in which the presentation is typically of a severe, drug-resistant, epileptic encephalopathy that is characterized by multiple seizure types, most notably gelastic seizures.^4^ Individuals with nonsyndromic HH may also exhibit precocious puberty, rage attacks, and neuropsychological comorbidities.^4^ Nonsyndromic HH arises sporadically without a family history of disease. Previous studies have identified postzygotic mosaic variants, most commonly in *GLI3*, in HH tissue resected from individuals with nonsyndromic HH.^5^, ^6^, ^7^, ^8^, ^9^, ^10^ To date, mosaic *GLI3* variants have not been detected in peripheral tissues despite longstanding suspicion that they might explain molecularly unsolved cases.^3^

We reasoned that individuals with PHS or nonsyndromic HH without an initial genetic diagnosis may harbor mosaic variants within *GLI3* below the level of detection of standard clinical genetic testing or not called by research variant calling pipelines that are designed to screen germline variants. Here, we interrogate high-depth exomes from peripheral blood leukocytes of 26 unsolved cases with PHS or nonsyndromic HH to increase genetic diagnostic yield and assess the contribution of blood mosaicism to *GLI3*-related HH.

## Materials and Methods

### Patient cohort

We studied 1 unsolved classical PHS case and 25 unsolved cases with apparent nonsyndromic HH who had drug-resistant epilepsy ascertained through epilepsy clinics.

### DNA extraction

DNA was extracted from whole blood using the QIAamp DNA Blood Maxi kit (Qiagen) or the Nucleon BACC Genomic DNA extraction kit (Cytiva). In 1 case with formalin-fixed paraffin-embedded (FFPE) HH tissue available from surgery, the Qiagen FFPE Tissue kit was used.

### Exome sequencing

A total of 25 individuals had exome sequencing with 200-fold coverage performed using the Agilent SureSelect DNA Human All Exon V6, 96RXN kit (Agilent Technologies) and the Illumina NovaSeq 6000 System (Illumina) with 150-bp paired-end reads. One individual (case 1) had exome sequencing targeting 100-fold coverage performed using the Agilent SureSelect XT Human All Exon + UTR V5 and the Illumina NovaSeq 6000 System with 100-bp paired-end reads. Briefly, reads were aligned to the hg19 reference genome with BWA-MEM v0.7.17-r1188, and then duplicate marking and base quality score recalibration were performed with the Genome Analysis Toolkit (GATK).^11^^,^^12^ Somatic variant calling was performed with GATK Mutect2 v4.0.1.2 using default parameters.^12^^,^^13^ Variants were annotated using vcfanno and ANNOVAR.^14^^,^^15^ Candidate variants were filtered to known or candidate cilia and SHH pathway genes compiled from the SYSCILIA Gold Standard and the Kyoto Encyclopedia of Genes and Genomes catalogs, which contain known HH-associated genes, including *CPLANE1*, *GLI3*, *OFD1*, and *SMO*.^16^^,^^17^ Variants were filtered according to the following criteria: located in a coding or splice site region, frequency of ≤0.0001 in the Genome Aggregation Database (gnomAD v2.1.1), and variant type—missense, nonsense, coding indel, or splice site.^18^ Coverage data were generated with mosdepth.^19^

### Droplet-digital PCR

Custom TaqMan SNP genotyping assays from Thermo Fisher Scientific Inc. were used to validate and quantify the mosaic *GLI3* variants. Droplet generation, polymerase chain reaction (PCR) cycling, and droplet reading were performed according to the manufacturer’s recommendations (Bio-Rad).

## Results

**Figure 1:**
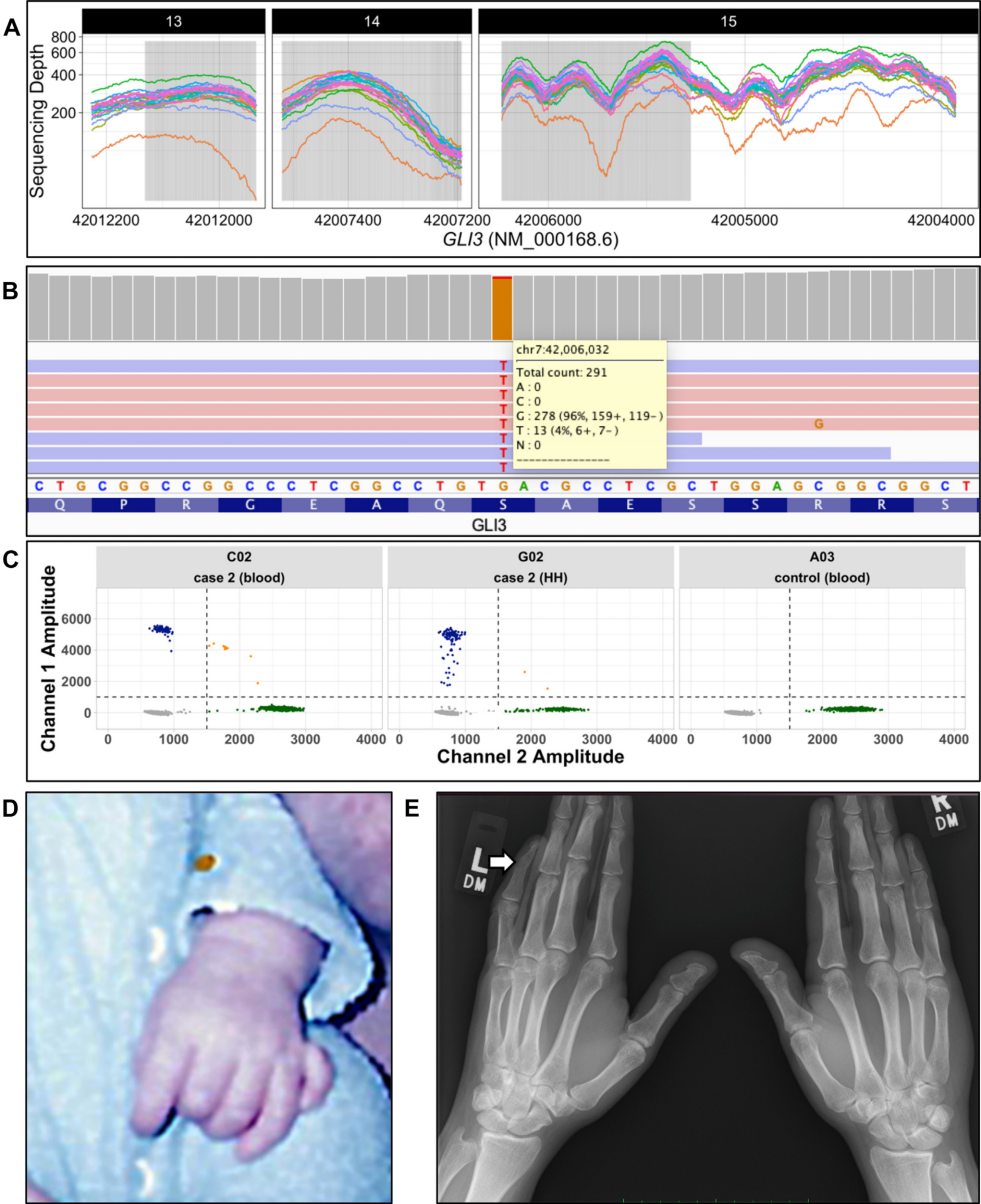
**Cohort-wide exome sequencing coverage of *GLI3* and clinical and molecular findings in case 2.** A. Exome sequencing coverage of all samples for exons 13, 14, and 15 of *GLI3* (NM_000168.6). The gray box represents the known PHS pathogenic region of *GLI3* (NM_000168.6: c.1998-3481) with a reported mean coverage of 324× across all samples. The lower orange sample with lower coverage represents case 1, which had exome sequencing with a target coverage of 100×. B. The Integrative Genomics Viewer screenshot shows the mosaic *GLI3* [NM_000168.6: 2639C>A; p.(Ser880Ter)] stop-gain variant in 13 of 291 (4.5%) sequencing reads of blood-derived DNA exome sequencing of case 2. Pink and blue colors indicate sequencing reads on the forward and reverse strands, respectively. C. The orthogonal validation using droplet digital PCR of the same variant in blood-derived and formalin-fixed paraffin-embedded HH-derived DNA samples at VAFs of 3.7% and 35.8%, respectively. Variant DNA copies (blue droplets), wild-type DNA copies (green droplets), droplets without DNA copies (gray droplets), and droplets with multiple DNA copies (orange droplets). The *x*-axis shows the amplitude of wild-type fluorescent probe; the *y*-axis shows the amplitude of variant-specific fluorescent probe. D. Photograph of case 2 from infancy showing left hand before surgical removal of a vestigial extra digit adjacent to his left fifth finger (postaxial polydactyly type B). E. Hand X-rays of case 2 at age 29 years, demonstrating symphalangism with fusion of middle and distal phalanges (white arrow). HH, hypothalamic hamartoma; PCR, polymerase chain reaction; PHS, Pallister-Hall syndrome; VAF, variant allele fraction.

All samples within our cohort had an exome-wide mean coverage greater than 100×, and the specific coverage of the PHS pathogenic region of *GLI3* (NM_000168.6: c.1998-3481) achieved a mean depth of 324× across the cohort, theoretically permitting detection of variant allele fractions (VAFs) of ≥4% at >99% sensitivity (Figure 1).^13^

### Patient with PHS

Case 1 is a 50-year-old man who had polydactyly of both hands and the right foot, which were surgically treated during childhood; left sided hemiatrophy, which caused facial asymmetry on the left; a shortened left arm and leg; and shortened left fifth finger. We could not examine the epiglottis, and we were unable to ascertain the type of polydactyly from historical medical records. He had onset of drug-resistant epileptic seizures at the age of 10 years, with an epigastric aura followed by gelastic seizures with oral and manual automatisms and aphasia. Focal to bilateral tonic-clonic seizures developed subsequently. Magnetic resonance imaging (MRI) revealed a large HH extending bilaterally and atrophy of the right hemisphere. Because of the size of the HH and the relatively mild phenotype with no encephalopathy, microsurgical resection was not recommended, and he declined radiosurgery. The patient reported 6 to 7 short gelastic seizures only per month. Neuropsychological testing revealed an IQ of 99, and he was fully employed. He had 2 healthy children.

Previous exome analysis had not revealed any candidate germline variants, including in *GLI3*. Visual inspection of the known PHS pathogenic region of *GLI3* (NM_000168.6: c.1998-3481) within aligned exome sequencing reads using the Integrative Genomics Viewer (IGV) revealed a *GLI3* stop-gain variant [NM_000168.6: c.2845G>T; p.(Glu949Ter)] in 10 of 165 (6.1%) sequencing reads of blood-derived DNA from case 1. Orthogonal validation by droplet-digital PCR (ddPCR) confirmed the VAF as 6.9% (Table 1).

**Table 1:** Clinical and genetic findings in individuals with mosaic *GLI3* variants

Case	Case 1	Case 2	Case 3
Clinical diagnosis	Pallister-Hall syndrome	Nonsyndromic HH	Nonsyndromic HH
Gender	Male	Male	Male
Age (y)	50	29	49
Gene	*GLI3*^a^	*GLI3*^a^	*GLI3*^a^
Variant	c.2845G>T; p.(Glu949Ter)	c.2639C>A; p.(Ser880Ter)	c.3326_3330del; p.(Glu1109AlafsTer18)
gnomAD v2.1.1 allele count	0	0	0
VAF ES blood (Alt/total reads)	6.1% (10/165)	4.5% (13/291)	10.4% (40/383)
VAF ddPCR blood	6.9%	3.7%	7.8%
VAF ddPCR HH	NT	35.8%	NT
Polydactyly	Polydactyly of both hands and right foot	Vestigial extra digit adjacent to his left fifth finger (postaxial polydactyly type B)	Nil
Craniofacial features	Left facial and left hemibody hypotrophy	Nil	Nil
MRI	HH, atrophy of the right hemisphere	HH	HH
Intellect	Normal	Mild ID, ASD	Mild ID, ASD
Seizures	Gelastic, tonic-clonic	Gelastic, tonic, FIAS	Gelastic, FIAS, tonic-clonic
Seizure onset age	10 y	2 mo	2 y

*ASD*, autism spectrum disorder; *ddPCR*, droplet-digital polymerase chain reaction; *ES*, exome sequencing; *FIAS*, focal impaired awareness seizure; *HH*, hypothalamic hamartoma; *ID*, intellectual disability; *MRI*, magnetic resonance imaging; *NT*, no tissue available; *VAF*, variant allele fraction.

a NM_000168.6.

### Nonsyndromic HH cases

We systematically interrogated *GLI3* and other HH-associated genes for mosaic variants in the remaining 25 unsolved cases with HH by applying the somatic variant calling algorithm Mutect2 (Materials and Methods).^12^ This variant call set revealed 2 cases who harbored different mosaic protein truncating variants (PTVs) in *GLI3*.

Case 2 is a 29-year-old man who presented with drug-resistant gelastic seizures at the age of 2 months. Subsequently, he developed tonic and focal impaired awareness seizures. Brain MRI revealed a HH. He had intellectual disability (ID), autism spectrum disorder (ASD), and behavioral comorbidities. Transcallosal HH resection was performed at the age of 4 years, as previously reported, and seizures markedly improved.^20^ He had a vestigial extra digit removed adjacent to his left fifth finger shortly after birth (postaxial polydactyly type B, Figure 1), which is usually considered an isolated, mild malformation of no clinical significance. Hand X-rays revealed no additional extra digits but did indicate symphalangism with fusion of the distal and middle phalanges of the left fifth finger (Figure 1). We could not examine the epiglottis, but he had no other features of PHS. Analysis of exome data revealed a *GLI3* stop-gain variant [NM_000168.6: c.2639C>A; p.(Ser880Ter)] in 13 of 291 (4.5%) sequencing reads (Figure 1, Table 1). We subsequently performed ddPCR to confirm the stop-gain *GLI3* variant at a VAF of 3.7% in blood (Figure 1, Table 1**)**. The HH tissue was preserved in formalin-fixed paraffin-embedded pathology slides, and ddPCR analysis showed that the variant was present at 35.8% VAF (Figure 1, Table 1).

Case 3 is a 49-year-old man who presented with drug-resistant gelastic seizures at the age of 2 years with later focal impaired awareness seizures and focal to bilateral tonic-clonic seizures. Brain MRI identified a HH. He had mild ID, ASD, IgA nephropathy, and behavioral comorbidities. No dysmorphic features were detected, and X-ray results of both hands and feet were normal. Analysis of blood-derived DNA showed a *GLI3* frameshift variant [NM_000168.6: c.3326_3330del; p.(Glu1109AlafsTer18)] in 40 of 383 (10.4%) sequencing reads. Independent validation by ddPCR confirmed that the variant was present at a VAF of 7.8% (Table 1). He has not undergone HH surgery.

## Discussion

We confirmed the genetic diagnosis in 3 unsolved cases with HH, 1 with PHS, and 2 with nonsyndromic HH, demonstrating that they harbored mosaic *GLI3* variants, which were detected in blood-derived DNA. To date, PHS and nonsyndromic HH caused by *GLI3* variants have been generally regarded as distinct entities because of divergent clinical presentations and underlying genetic architecture, the former germline and the latter mosaic in the brain. The clinical diagnosis of PHS has historically been stringent with the requirement of HH and mesoaxial polydactyly driving collection of homogeneous cohorts in initial gene discovery efforts.^2^^,^^21^ Over time, the diagnostic criteria for PHS have slowly expanded to include cases with nonclassical PHS, including, rarely, patients without polydactyly.^21^ As in case 1, epilepsy in PHS is generally mild or absent.^3^^,^^22^ In contrast, nonsyndromic HH usually presents with drug-resistant gelastic and other seizures, sometimes with ID, ASD, behavior disturbance, and a progressive course reflecting an epileptic encephalopathy. The difference in the presentations may reflect referral bias, and mild cases of nonsyndromic HH are recognized.^23^

Previous genetic analyses of individuals with *GLI3*-related HH suggests that a subset of nonsyndromic HH may be etiologically similar to PHS. Preceding studies have demonstrated a robust genotype-phenotype correlation for PHS, with germline PTVs in the middle third of the *GLI3* gene in most cases.^2^^,^^24^ This uniform gene and variant spectrum in individuals with PHS has permitted a high genetic diagnostic rate (∼95%).^2^ The etiology of nonsyndromic HH is genetically heterogeneous with the majority of cases remaining unsolved, likely owing to the difficulty in mosaic variant detection and access to HH tissue. However, to date, approximately 15% of patients with nonsyndromic HH have mosaic *GLI3* variants in sequenced HH tissue.^6^, ^7^, ^8^^,^^10^ All of these reported mosaic *GLI3* variants are PTVs, residing in the pathogenic PHS region of *GLI3*.^6^, ^7^, ^8^^,^^10^ Until now, the genetic findings in *GLI3-*related HH have been demarcated by germline variants in PHS, and mosaic variants presumed to be restricted to brain in individuals with nonsyndromic HH.

In line with increased recognition of mosaicism underlying human disease more broadly, our new findings reported here suggest that mosaicism in *GLI3*-related HH disorders should be considered a spectrum ranging from variants being confined to the brain (and possibly only the hypothalamus) to being present more widely in the body and detectable, albeit at low level, in peripheral leukocytes. Furthermore, the corresponding phenotypic variability may be in part explained by the origin and varying tissue distribution of the variant.

Specifically, the 3 mosaic *GLI3* PTVs detected were all present at low VAFs in blood-derived DNA (Table 1, Figure 1). All 3 variants are novel in the Genome Aggregation Database (gnomAD v2.1.1) and follow the established PHS variant spectrum being PTVs in the middle region of *GLI3* (NM_000168.6: c.1998-3481).^18^ In case 2, we confirmed that the *GLI3* variant was present in his resected HH tissue at about 10-fold greater VAF than in his blood-derived DNA (Figure 1). This suggests that the mosaic *GLI3* variants likely arose early in development before gastrulation and germ layer differentiation given the presence of the variants in the blood and central nervous system. Although the 3 individuals we report here share similar VAFs of their respective mosaic *GLI3* variants, there is significant phenotypic variability, ranging from PHS to nonsyndromic HH without additional PHS stigmata. This implies that the functional impact of the underlying variant is also likely to affect the clinical presentation, perhaps influenced by the individual’s genetic background. In case 1, MRI confirmed atrophy of the right hemisphere, which, to our knowledge, has not been associated with PHS; therefore, it is unclear whether this feature is related to the mosaic *GLI3* variant identified in this individual. Further delineation of *GLI3* genotype-phenotype correlations is required to understand these relationships, including from the most severe (fetal) cases of PHS.^21^

Our findings indicate that individuals with HH, with or without features of PHS, may harbor blood mosaicism of *GLI3* variants previously undetected in other studies.^21^ One previously reported family with 2 children affected with PHS, but with unaffected parents is suspected to have arisen through gonadal mosaicism.^25^ Importantly, our findings confirm the long-standing hypothesis that PHS can arise through postzygotic mosaicism within *GLI3*.^3^ These data will provide the impetus to apply more sensitive genetic screening techniques to those individuals with suspected PHS to exclude mosaicism in *GLI3* and potentially other SHH pathway or cilia genes.^7^^,^^10^ The identification of mosaicism will inform new clinical testing guidelines that extend beyond germline variation and guide genetic counseling of affected individuals and their families. Most importantly, our findings extend the *GLI3*-related Pallister-Hall spectrum and provide data to support extending the investigation of mosaicism beyond pathogenic lesions to peripheral tissues such as blood in other Mendelian disorders.

## Data Availability

The identified variants are publicly available in the Leiden Open Variation Database.

## Conflict of Interest

The authors declare no conflicts of interest.
